# Supplementary material for: An equation-of-state-meter of quantum chromodynamics transition from deep learning
Source: Nat Commun. 2018 Jan 15;9:210. doi: 10.1038/s41467-017-02726-3 (PMC5768690; doi:10.1038/s41467-017-02726-3)
Supplement: Supplementary file 1 — Supplementary information [file 41467_2017_2726_MOESM1_ESM.pdf]

SUPPLEMENTARY INFORMATION

Supplementary Note 1, basics of deep neural network

Feedforward neural network learns one target function $\mathbf{x} : f(\mathbf{x}, \theta) \rightarrow \mathbf{y}$ that maps the input vector $\mathbf{x}$ to output vector $\mathbf{y}$ with parameter θ. Elements of $\mathbf{x}$ and $\mathbf{y}$ form the neurons in the input and output layers respectively. In-between there can be multiple hidden layers with the numbers of neurons as hyper-parameters. The connections between two layers form a trainable weight matrix $\mathbf{W}$. Each layer (except the input layer) learns representations of its previous layer through firstly a linear operation $\mathbf{z} = \mathbf{x}\mathbf{W} + \mathbf{b}$ and then use it as the argument of an activation function $\sigma(\mathbf{z})$. The linear operation can perform various operations, such as scaling, rotating, boosting, increasing or decreasing dimensions, on the vector $\mathbf{x}$, with the bias $\mathbf{b}$ a trainable parameter. $\sigma(\mathbf{z})$ activates the neurons of the present layer with their values and computes the correlations between the neurons of the previous layer. For classification network, softmax activation function $\sigma(\mathbf{z})_j = \exp(z_j)/\sum_{k=1}^{K} \exp(z_k)$ is usually used in the final layer to compute the probability of each category. By stacking with multiple hidden layers, the deep neural network may learn high-level representations that can be classified or interpreted easily. The activation functions used in our study are shown in Supplementary Figure 1.

Loss function $l(\theta)$ is the difference between the true value $\mathbf{y}$ (from the input of supervised learning) and the predicted value $\hat{\mathbf{y}} = f(\mathbf{x}, \theta)$ by the neural network in a forward pass. The simplest loss function is the mean square error $l(\theta) = \sum_i (\hat{y}_i - y_i)^2$. In this paper we use the cross entropy loss function from information theory,

$$l(\theta) = -\frac{1}{N} \sum_{i=1}^{N} \left[y_i \log \hat{y}_i + (1 - y_i) \log(1 - \hat{y}_i) \right] \tag{1}$$

With L1 or L2 regularizations, the loss function receives another term used to constrain the values of θ from going wildly,

$$L1 : \; l(\theta) = l(\theta) + \lambda ||\theta||_1 \tag{2}$$

$$L2 : \; l(\theta) = l(\theta) + \lambda ||\theta||_2^2 \tag{3}$$

where λ is the regularization strength, $||\theta||_p \equiv \left(\sum_j^n |\theta_j|^p \right)^{1/p}$ is the p-norm of the parameters $\theta = (\theta_1, \theta_2, ..., \theta_n)$. Larger λ leads to smaller θ, especially for high orders in the target function, which increases the generalizability of the neural network.

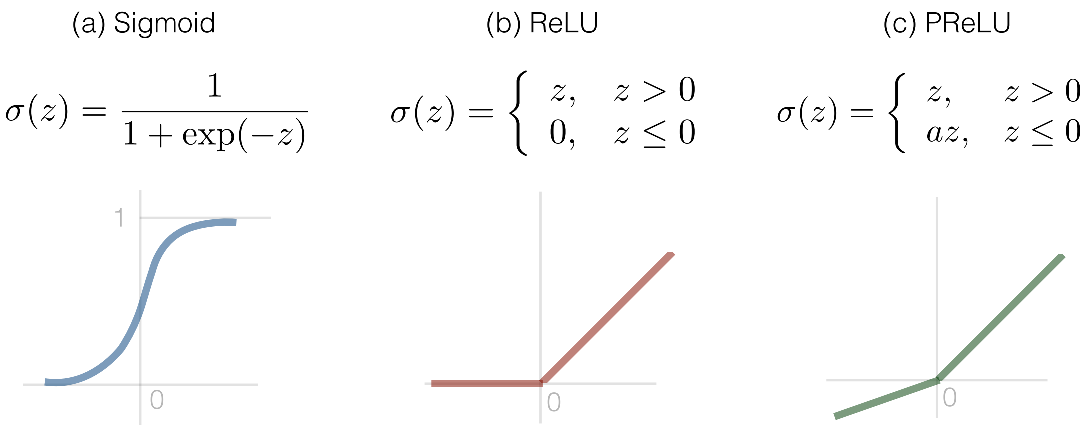

Supplementary Figure 1. Activation functions related to the present study. (a) Sigmoid, the logistic function which has a S shaped curve (b) ReLU, rectified linear unit that activates the neuron when $z > 0$ and (c) PReLU parametric rectified linear unit that additionally activates leaky neurons at $z < 0$ with learnable parameter a.

Back propagation indicates the gradients of the loss function in parameter space propagate in the backward direction of a neural network in order to update θ. For example, in the stochastic gradient decent (SGD) method, θ is updated with fixed learning rate ϵ

$$\theta^{'} = \theta - \epsilon \frac{\partial l(\theta)}{\partial \theta} \tag{4}$$

In practice we train the network in batches, where θ is updated once for all the samples in one batch,

$$\theta^{'} = \theta - \frac{\epsilon}{m} \sum_{i=1}^{m} \frac{\partial l_i(\theta)}{\partial \theta} \tag{5}$$

where m is the batchsize, l_i is the loss given by the ith training sample in a batch. In our study, we use the AdaMax method [1], which computes adaptive learning rates for different parameters based on estimating the first and second moments of the gradients. We initially set the learning rate as $\alpha = 10^{-4}$ and keep the other parameters the same as in [1].

Batch normalization solves the internal covariate shift problem, which is a common issue in DL that hinders the learning efficiency [2]. Using the batch mean $\mu_B = \frac{1}{m}\sum_{i=1}^{m} x_i$ and batch variance $\sigma_B^2 = \frac{1}{m}\sum_{i=1}^{m}(x_i - \mu_B)^2$, the input vector $\mathbf{x}$ is normalized as $\hat{x}_i = \frac{x_i - \mu_B}{\sqrt{\sigma_B^2 + \epsilon}}$ that has mean 0 and variance 1, with ϵ a small number preventing divergence. The $\hat{\mathbf{x}}$ is further scaled and shifted by $\gamma\hat{\mathbf{x}} + \beta$ before going to the next layer, where γ and β are trainable

parameters. Note that during the testing, population mean and variance of the training dataset are used.

Dropout is a regularization technique that reduces overfitting by randomly discarding a fraction of neurons (features) and all their associated connections to prevent co-adaption [3] of neurons for each training sample .

Prediction Difference Analysis is a method to visualize the difference between the log-odds of the prediction probability $p(y|\rho)$ and $p(y|\rho_{\setminus i})$, where y is the class value, ρ is the real image and $\rho_{\setminus i}$ is the imperfect image without the knowledge of the ith pixel. The prediction difference is dubbed as weight of evidence [4, 5],

$$\mathrm{WE}_i(y|\rho) = \log_2 \left(odds(y|\rho)\right) - \log_2 \Big(odds(y|\rho_{\setminus i})\Big) \ , \tag{6}$$

where $odds(z) = p(z)/(1-p(z))$ is used to symmetrize $\log_2 p$ and $-\log_2(1-p)$, with Laplace correction $p \leftarrow (pn+1)/(n+m)$ to avoid zero probability, where n is the number of training instances and m is the number of classes. The $p(y|\rho_{\setminus i})$ is approximated by,

$$p(y|\rho_{\setminus i}) \approx \sum_{s}^{m_i} p(\rho_i) p(y|\rho \leftarrow \rho_i = a_s) \, , \tag{7}$$

with the ith pixel replaced with all the possible values a_s weighted by its value probability. The importance map is given by the mean weight of evidence over many events that have the same class label.

Supplementary Note 2, results from traditional machine learning methods

Many observables are designed from the raw spectra $\rho(p_\mathrm{T}, \phi)$ during the last two decades. The most widely applied observables to constrain QGP properties are the transverse momentum distributions and the Fourier decomposition coefficients v_n along the azimuthal angle direction. We computed 85 such observables – mean transverse momentum $\langle p_\mathrm{T} \rangle$, multiplicity dN/dY at mid-rapidity (rapidity $Y = 0$), p_T integrated v_n, event plane angles ψ_n, and $v_n(p_\mathrm{T})$ at 15 different p_Ts for n in one of the $2, 3, 4, 5$. As shown in Supplementary Figures 2, 3 and 4, the event-by-event distribution of a few important observables – $\langle p_\mathrm{T} \rangle$, v_n and event angles ψ_n – do overlap for 2 different EoS cases and can not be distinguished by the traditional methods developed in high-energy heavy-ion science.

Note that for the training data from CLVisc+AMPT Monte Carlo model, the magnitude of the initial entropy densities is adjusted such that relativistic hydrodynamics with two different EoSs produces degenerate results on the traditional observables. While for the testing data from the CLVisc+IP-Glasma model, hydrodynamics starting with equal initial conditions and freeze-out temperatures produces more particles and higher $\langle p_{\mathrm{T}} \rangle$ for the EOSQ case due to longer lifetime. The effect of shear viscosity in the CLVisc+IP-Glasma model is also distorted by employing different longitudinal distributions for EOSL and EOSQ. These setups are made to prevent over-fitting to one specific group of model parameters. Deep CNN trained with data from CLVisc+AMPT model (the first column) has very good generalizability on data from the iEBE-VISHNU (the second column) and CLVisc+IP-Glasma (the third column) models, as shown in Supplementary Figures 2, 3 and 4.

The correlation matrix of pairs of important distinct observables is shown in Supplementary Figure 5, The correlation matrix shows the strength of big data analysis for the simulated data. The results are shocking – correlations between various observables are revealed by a few lines of python codes. It took several years of effort for high energy physicists to find some of these correlations, one after another: the strong correlation between v_2 and v_4 had been known for more than 10 years at the RHIC [6]. The strong correlations between v_2 and v_5, v_3 and v_5 have been only verified quite recently at the LHC [7]. The data show negative correlations between v_2 and dN/dy, and almost zero correlation between v_2 and $\langle p_{\mathrm{T}} \rangle$. These results are physically understandable. The strong positive correlation between $\langle p_{\mathrm{T}} \rangle$ and v_5 is a new type of correlation which has not been discovered before. Scientists in the heavy-ion community usually only use the mean value of these observables or the correlation between these observables searching for constraints of the properties of the hot and dense quantum chromodynamics matter.

Concerning traditional machine learning tools, we have systematically checked the prediction accuracies with various classifiers using scikit-learn. As shown in Supplementary Table 1, the best performance (with good generalization capability) is obtained by a linear support vector machine classifier (linear-SVC) – on average 80% prediction accuracy using the best estimator from a grid search on pre-defined observables. The ensemble methods of random forest and gradient boosting trees get higher score than a single decision tree. Gaussian Naive Bayes classifier and pca + linear SVC have poor generalization capability on two groups of testing data – with less than 50% accuracy.

Prediction Accuracies	GROUP1	GROUP2
obs + Gaussian Naive Bayes	46.2%	47.6%
obs + Decision Tree	57.5%	64.9%
obs + Random Forest	62.5%	69.8%
obs + Gradient Boosting Trees	66.9%	81.9%
obs + linear SVC	75.8%	84.6%
obs + SVC rbf kernel	60.9%	56.7%
raw + linear SVC	65.2%	84.3%
pca + linear SVC	46.4%	47.7%

Supplementary Table 1. The prediction accuracies of traditional machine learning algorithms. Prediction accuracies from Gaussian Naive Bayes, Decision Tree, Random Forest, Boosting Trees and Support Vector Machine (SVC) using scikit-learn [8]. Where obs stands for 85 pre-defined observables, raw stands for raw spectra $\rho(p_{\rm T}, \phi)$ and pca denotes the first 150 components from principle component analysis (PCA) on raw spectra.

For the trained linear-SVC classifier with pre-defined observables, we list the 20 most important features in descending order – ptspec-bin4, ptspec-bin5, ptspec-bin8, ptspec-bin7, ptspec-bin6, ptspec-bin1, dN/dy, ptspec-bin2, ptspec-bin3, ptspec-bin11, v2-ptbin5, v2-ptbin6, v2-ptbin4, ptspec-bin9, v5-ptbin12, ptspec-bin10, v5-ptbin11, ptspec-bin12, ptspec-bin0, v2-ptbin7. Apparently both the shape of the $p_{\rm T}$ spectra and the azimuthal angle distribution at different $p_{\rm T}$s dominated by the bins 4 - 8 (correspond to $p_{\rm T}$ range $0.3 \sim 1.0$ GeV) are decisive for the classification capability of this linear-SVC. Trained with the pre-defined observables, the shape of the $p_{\rm T}$ spectra is more important than the azimuthal angle distribution. The important $p_{\rm T}$ range obtained from the linear-SVC agrees with the importance map from the prediction difference analysis of the deep convolution neural network.

The important features of this linear-SVC trained with raw spectra is shown in the Supplementary Figure 6. The $p_{\rm T}$ range in this importance map covers part of that of the deep CNN. However, the most important features along the azimuthal angle direction are centered at $\phi = \pi/2$ and $\phi = 3\pi/2$. Since we want to confirm that the dynamical evolution is indeed encoded in the final state particle spectra, a deep neural network works much better: the prediction accuracy is much higher and the importance region follows intuition.

Our analysis shows that not only deep learning, but also some traditional big data analysis with machine learning tools now bring novel insights to the heavy-ion community, provided relativistic heavy-ion collisions have accumulated a huge amount of data.

---

[1] D. Kingma and J. Ba, “Adam: A Method for Stochastic Optimization”, https://arxiv.org/abs/1412.6980 (2014).

[2] S. Ioffe and C. Szegedy, “Batch Normalization: Accelerating Deep Network Training by Reducing Internal Covariate Shift”, https://arxiv.org/abs/1502.03167 (2015).

[3] G. E. Hinton, N. Srivastava, A. Krizhevsky, I. Sutskever, and R. R. Salakhutdinov, “Improving neural networks by preventing co-adaptation of feature detectors”, https://arxiv.org/abs/1207.0580 (2012).

[4] M. Robnik-Sikonja and I. Kononenko, “Explaining classifications for individual instances”, Knowledge and Data Engineering, IEEE Transactions on, 20(5):589-600, (2008).

[5] L. M. Zintgraf, T. S. Cohen, T. Adel, and M. Welling, “Visualizing deep neural network decisions: prediction difference analysis”. https://arxiv.org/abs/1702.04595 (2017).

[6] J. Adams *et al.* [STAR Collaboration], “Azimuthal anisotropy in Au+Au collisions at s(NN)**(1/2) = 200-GeV,” Phys. Rev. C **72**, 014904 (2005).

[7] G. Aad *et al.* [ATLAS Collaboration], “Measurement of the correlation between flow harmonics of different order in lead-lead collisions at $\sqrt{s_{NN}}$=2.76 TeV with the ATLAS detector,” Phys. Rev. C **92**, no. 3, 034903 (2015).

[8] Pedregosa et al., “Scikit-learn: Machine Learning in Python”, Journal of Machine Learning Research, **12**, 2825-2830 (2011).

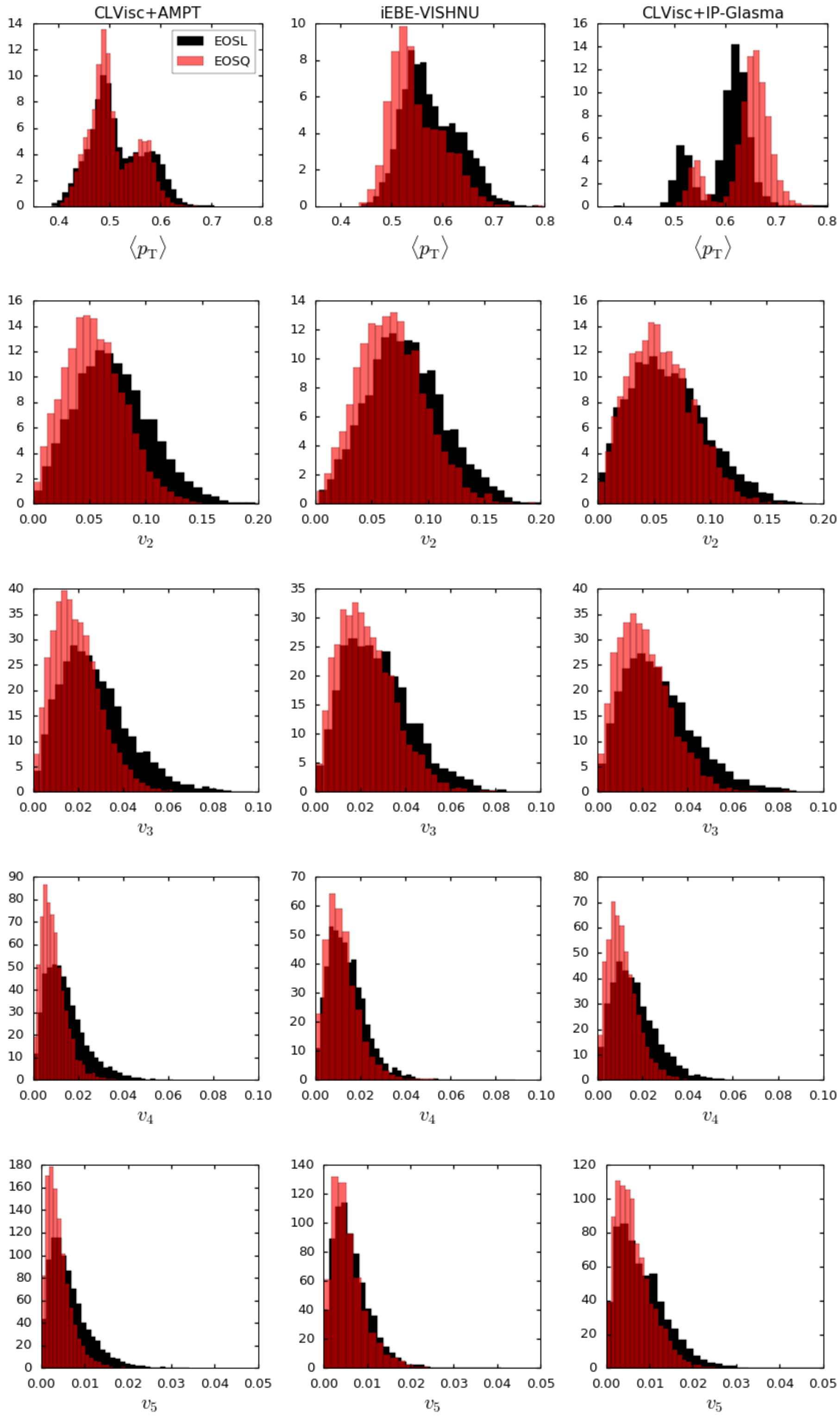

Supplementary Figure 2. The event-to-event distribution of mean $p_{\rm T}$ and v_n. The distribution of $\langle p_{\rm T} \rangle$ (the first row) and v_n (n=2, 3, 4, 5 for the nth row) for the CLVisc+AMPT (first column), the iEBE-VISHNU+MCGlauber (the second column) and the CLVisc+IP-Glasma (the third column) models.

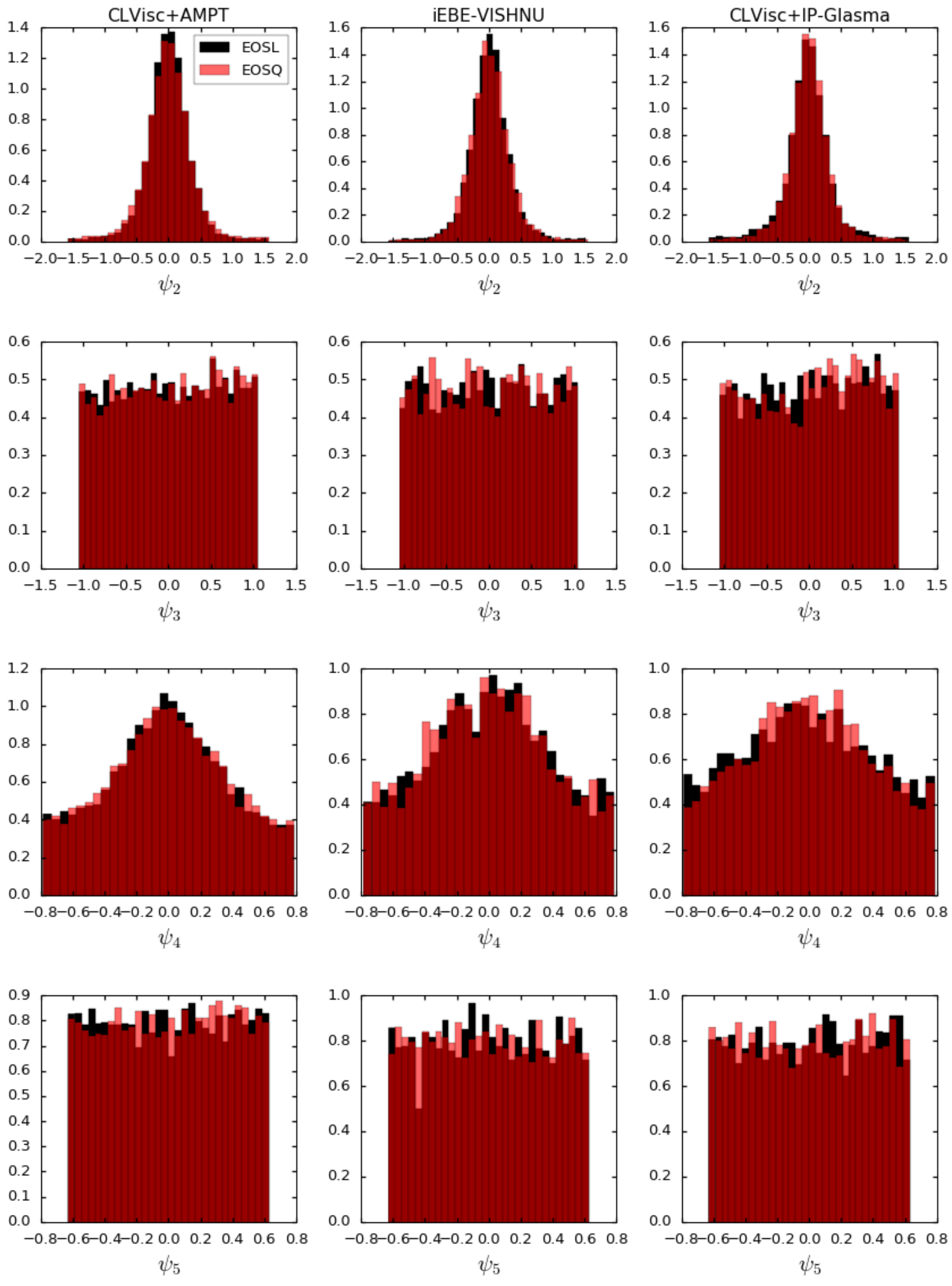

Supplementary Figure 3. The event-to-event distribution of event planes. The distribution of ψ_2 (the first row), ψ_3 (the second row), ψ_4 (the third row) and ψ_5 (the fourth row) for the CLVisc+AMPT (first column), the iEBE-VISHNU (the second column) and the CLVisc+IP-Glasma (the third column) models.

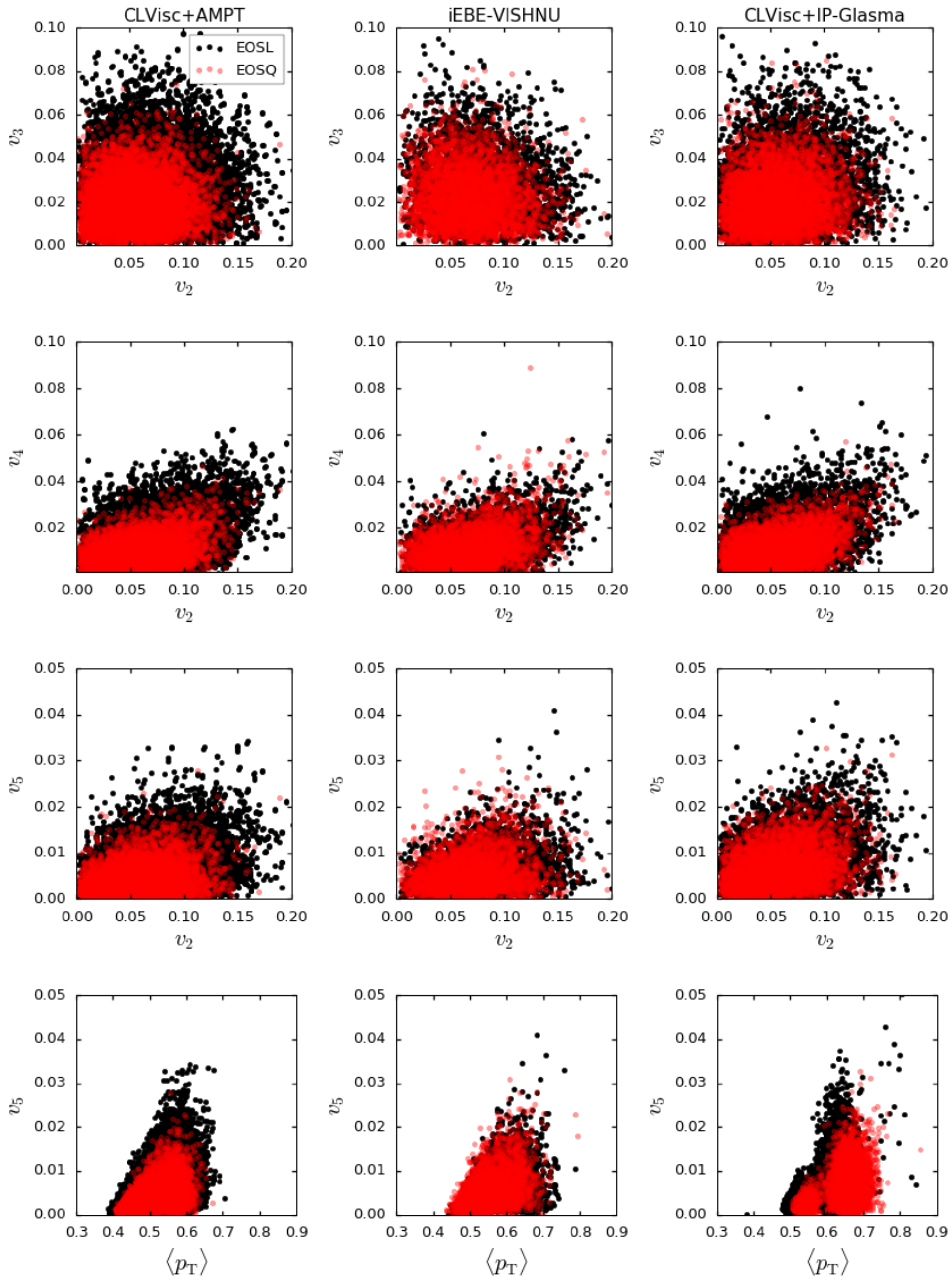

Supplementary Figure 4. The scatter plots between several pairs of observables. The scatter plots between v_2 and v_3 (the first row), v_2 and v_4 (the second row), v_2 and v_5 (the third row), $\langle p_\mathrm{T} \rangle$ and v_5 for the CLVisc+AMPT (first column), the iEBE-VISHNU (the second column) and the CLVisc+IP-Glasma (the third column) models.

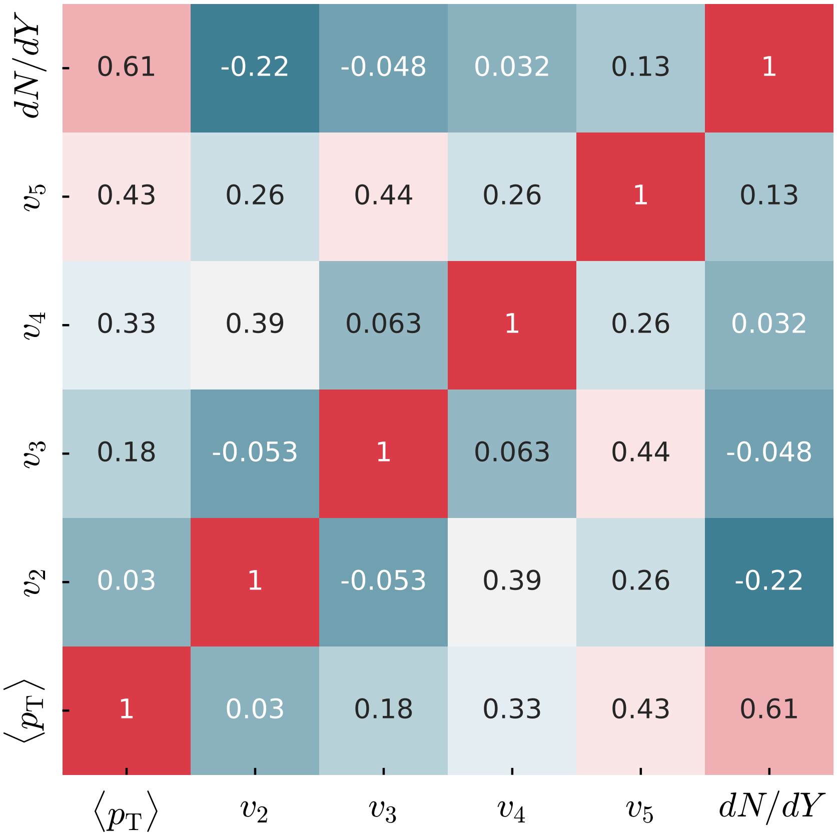

Supplementary Figure 5. The correlation matrix between many observables. The correlations matrix between $\langle p_{\mathrm{T}} \rangle$, $v2$, $v3$, $v4$, $v5$ and dN/dY on testing data GROUP1, reveals various correlations that were found one after another in the last two decades.

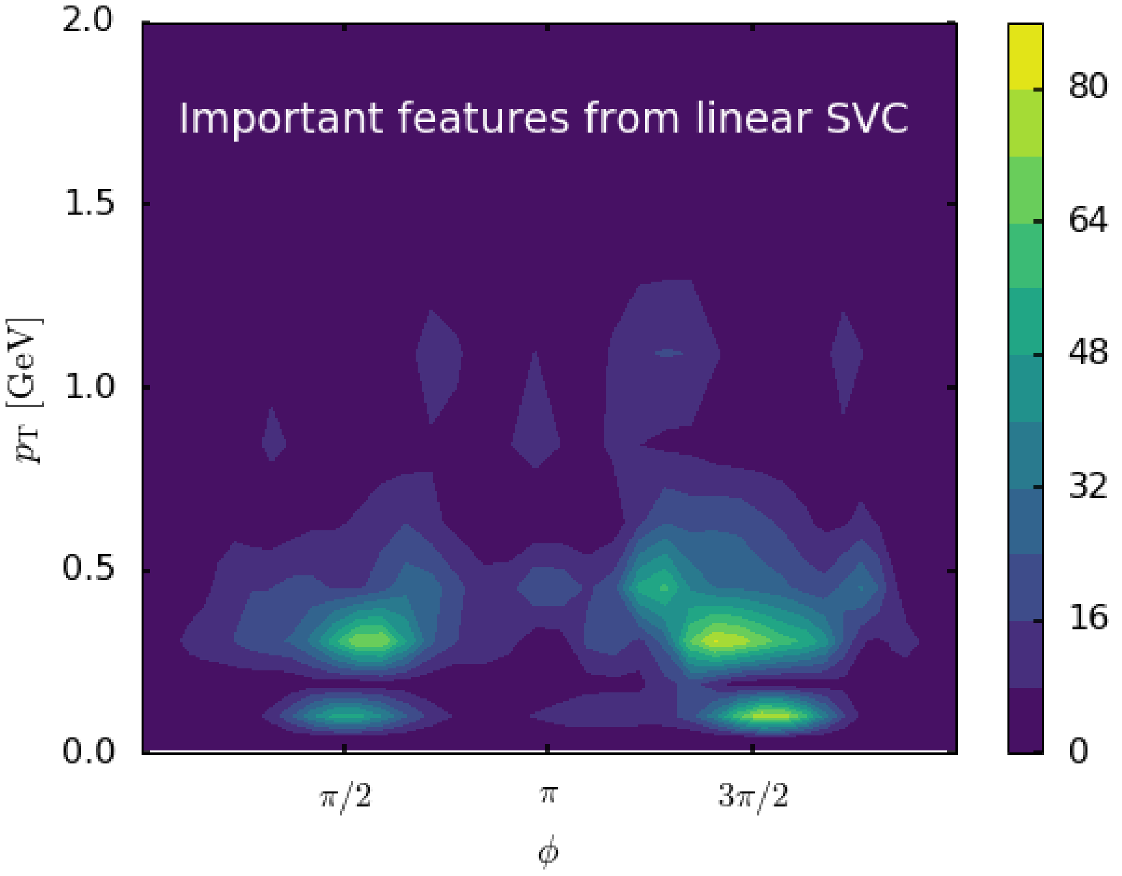

Supplementary Figure 6. The important features from linear support vector machine. The linear support vector machine is trained with raw spectra $\rho(p_T, \phi)$ and the intensity of this importance map is given by the square of the linear support vector machine weights.
